# Adrenal steroid hormone responses to exercise under thermal stress: Potential role for nonclassic congenital adrenal hyperplasia in heat illness susceptibility

**DOI:** 10.14814/phy2.70272

**Published:** 2025-03-20

**Authors:** Michael J. Stacey, Carol House, Daniel Roiz de Sa, Stephen J. Brett, Christopher Boot, Andrew Teggert, Adrian J. Allsopp, David R. Woods

**Affiliations:** ^1^ Department of Military Medicine Royal Centre for Defence Medicine Birmingham UK; ^2^ Department of Surgery and Cancer Imperial College London London UK; ^3^ Carnegie School of Sport Leeds Beckett University Leeds UK; ^4^ Environmental Medicine and Science Division Institute of Naval Medicine Gosport UK; ^5^ General Intensive Care Unit Hammersmith Hospital London UK; ^6^ Blood Sciences Newcastle Upon Tyne Hospitals NHS Foundation Trust Newcastle Upon Tyne UK; ^7^ Clinical Biochemistry South Tees Hospitals NHS Foundation Trust Middlesbrough UK

**Keywords:** dehydration, heatstroke, hyponatremia

## Abstract

We queried whether adrenal insufficiency attributable to non‐classic congenital adrenal hyperplasia (21 hydroxylase deficiency, 21OHD) might contribute to heat illness susceptibility. Patients referred to a specialist heat illness clinic (*n* = 2 with prior hyponatremia; *n* = 16 lacking documentary evidence) and controls (*n* = 16) underwent laboratory Heat Tolerance Assessment (HTA: 60–90 min walking, 60% relative intensity, 34°C heat), synthetic adrenocorticotrophic hormone stimulation (heat illness only) and CYP21A2 genotyping (hyponatremic heat illness only). Copeptin, cortisol, 17‐hydroxyprogesterone, and 21 deoxycortisol were assayed from blood at baseline and post‐HTA, with precursor product [17‐hydroxyprogesterone +21 deoxycortisol] expressed relative to cortisol. Saliva and urine were assayed for free cortisol (one hyponatremic case, controls). Versus controls, normonatremic heat illness exhibited greater (*p* < 0.05) serum cortisol across HTA, while hyponatremic heat illness showed blunted responses in aldosterone and free cortisol (salivary cortisol 1.6 and 1.6 vs. 6.0 [4.2, 19.4] and 4.2 [3.8, 19.2] nmol.L‐1; urine cortisol 19 vs. 117 +/− 71 nmol.L‐1). Hyponatremic heat illness demonstrated elevated precursor product consistent with 21OHD and multiple CYP21A2 mutations. One normonatremic case of heat illness also showed elevated precursor product. These data support the potential for 21OHD to precipitate heat illness under sustained physical stress and advance a case for targeted genetic screening.

## 1 INTRODUCTION

A hallmark of the human species is its remarkable phenotypic plasticity in the face of increased thermal challenge (Notley et al., 2024). Nevertheless, substantial interindividual variability in thermal tolerance is recognized (Corbett et al., 2018).

Heat‐related illnesses, including fatal heat stroke, may be complicated by a fall in serum levels of sodium below the recognized laboratory reference range (typically <135 mmol/L) (Backer et al., 1999; Miyasaka et al., 2013; Nolte et al., 2015; Oh et al., 2018; Sankar et al., 2020; Satirapoj et al., 2016). This phenomenon of incapacity precipitated primarily by thermal stress (“heat illness”) coexisting with hyponatremia has been reported across a range of populations, including military personnel (Nolte et al., 2015; Oh et al., 2018; Sankar et al., 2020), recreational and professional athletes (Backer et al., 1999), and workers in agricultural and industrial settings (Miyasaka et al., 2013; Satirapoj et al., 2016; Wesdock & Donoghue, 2019). Extremes of climatic stress are implicated, with rates of intercurrent hyponatremia as high as 75% in Indian military recruits affected by heat illness (Sankar et al., 2020), 50% in exertional heat stroke cases admitted to hospital in Thailand (Satirapoj et al., 2016), and 48% in classic heatstroke cases presenting to the emergency department during a heatwave (Argaud et al., 2007). The phenomenon was also observed in large numbers of United Kingdom Service Personnel deployed into the extreme heat of Kuwait and Iraq at the beginning of the century (Coleman et al., 2022; Day & Grimshaw, 2005; Grainge & Heber, 2005; Hamilton et al., 2006; World & Booth, 2008).

Cases of exertion‐related hyponatremia have been associated more commonly with overdrinking of solute‐poor fluids during physical activity (Katch et al., 2017), whereby the development of encephalopathy from low serum sodium is implicated in reduced thermal awareness and heightened risk of concomitant heat illness (Miyasaka et al., 2013; Nolte et al., 2015). While other extrinsic factors have also been identified and investigated systematically (e.g., nonsteroidal use) (Fitzpatrick et al., 2024), the attention paid to intrinsic risk factors has been comparatively less and has centered largely on extremes of sodium losses from high rates of thermal sweating and/or sweat sodium concentration (Meyer et al., 2015). As demonstrated originally by Jerome Conn, patients affected by primary hypoadrenalism show a 50‐to‐60‐fold greater concentration of sodium in sweat than subjects with adequate aldosterone responses (Conn, 1949). Thus, it may be argued that occult adrenal insufficiency should be considered in heat illness, especially those instances arising under prolonged physiological stress where hyponatremia is a complicating factor.

Among the possible candidates for genetic susceptibility of this nature, congenital adrenal hyperplasia (CAH) in its non‐classic, late‐presenting form is worthy of suspicion. With a reported prevalence of 1:1000 to 1:100 (Hannah‐Shmouni et al., 2017; White & Speiser, 2000), non‐classic CAH (NCCAH) has been considered a highly common genetic polymorphism (Speiser et al., 1985), with a carrier frequency of up to 1 in 10 in Europe (Baumgartner‐Parzer et al., 2005). In females, NCCAH presents typically with reproductive disturbances that may be confused for other common pathologies, whereas it is very rarely identified in men, who may remain asymptomatic (Speiser et al., 1985). The majority of NCCAH encountered in clinical practice is attributable to mutations in the gene CYP21A2 causing 21‐hydroxylase deficiency (21OHD) (Krone et al., 2007). Depending on the nature of mutations, homozygotes for CYP21A2 may retain 40%–60% of functionality in 21‐hydroxylase, whereas in heterozygotes the presence of one normal allele limits but may not entirely abolish enzymatic deficiency, belying the autosomal nature of the condition. NCCAH is conventionally characterized by an absence of adrenal crises (Glass et al., 1994; Jha & Turcu, 2021), however, apparent dependency on exogenous steroids has been described previously in a military serviceperson (Glass et al., 1994), in whom clinical and metabolic disturbances resolved only with their administration.

Based upon observations of subnormal glucocorticoid adrenal function and relative defect in mineralocorticoid production in heterozyygotes (Fiet et al., 1989), it has been suggested that transient glucocorticoid supplementation and rehydration be considered during periods of acute stress, particularly in those individuals shown to have bi‐allelic mutations (Kamenický et al., 2019). Specifically, half of females with NCCAH showed partial cortisol deficiency with insulin tolerance testing, and plasma renin concentrations around 80% higher than controls subject to equivalent sodium depletion over 24 h (Kamenický et al., 2019), a so‐called “compensated partial aldosterone insufficiency.”

Nevertheless, NCCAH, despite being one of the most common autosomal recessive disorders (Baumgartner‐Parzer et al., 2005; Hannah‐Shmouni et al., 2017; Speiser et al., 1985; White & Speiser, 2000), remains a challenge to identify and diagnose, with genetic testing often positive in the face of seemingly reassuring biochemical results. Specifically, measures of cortisol and aldosterone may appear to indicate satisfactory adrenal reserve, while their steroid hormone precursors—which classically accumulate with CAH due to shunting across into the androgen pathway—may also fail to provide a reliable indication of the condition. However, the sum of the precursor 17‐hydroxyprogesterone and its metabolite 21‐deoxycortisol, relative to the downstream cortisol product, that is, ((17OHP + 21DF)/F), has been reported to show a discriminant increase as the phenotype progressively develops from unaffected controls to “manifesting heterozygotes” (Witchel et al., 1997), to NCCAH (Ng et al., 2023).

In order to explore whether a history of heat illness would be associated with evidence for 21OHD, this study aimed to characterize adrenal steroid hormone responses to thermal stress in individuals diagnosed with heat illness, including those with documented hyponatremia, and in healthy controls exposed to comparable exercise in hot conditions. Secondary aims included exploration of endocrine responses between cases and controls, such as variation in the release of the vasopressin surrogate copeptin; testing of adrenal reserve with synthetic adrenocorticotropin (short synACTHen test); and genetic investigation for NCCAH.

## 2 METHODS

### 2.1 Participants

Patients were recruited from sequential referrals to the United Kingdom military Heat Illness Clinic (HIC), a specialist service provided to risk stratify heat illness cases complicated by significant organ or tissue injury for safe return to duties. Heat illness cases with no record of biochemical hyponatremia concomitant with their heat illness episode (normonatremic Heat Illness, nHI, *n* = 16) performed an exercising Heat Tolerance Assessment (HTA) at least 8 weeks' following their incapacitation from heat illness, a period in which they were protected from thermal stress. This procedure, a clinical standard of care at the clinic, is conducted in order to interrogate the ability to maintain thermal equilibrium in physiologically compensable conditions, in the unacclimatised state (House et al., 2023).

The nHI cohort was compared with two historical cases of hyponatremic heat illness (hHI1 and hHI2) assessed previously through the clinic, both of whom were recruited to repeat HTA and complete other study measures.

A further cohort (*n* = 16) of healthy military volunteers was recruited, with no history of heat illness and/or hyponatremia (controls). No restrictions were placed upon prior physical activity or climatic exposure in these individuals. All control participants were based in the United Kingdom. Assessments were performed between May and July in the same year.

Ethical approval was obtained from the UK Ministry of Defence Research Ethics Committee (856/MODREC/2018), and the study was conducted in accordance with the Declaration of Helsinki, with written informed consent provided by all participants. All individuals participating in the study were required to pass a medical examination on the day of their participation, including completion of a health history questionnaire, 12‐lead electrocardiogram, blood pressure, and clinical examination by the HIC medical officer.

### 2.2 Outline and experimental measures

A large environmental chamber was used for the determination of peak exercising oxygen uptake (VO_2_peak) and subsequent HTA. With the exception of exercise testing in the environmental chamber, preparation, rest, and recovery of participants was undertaken in an adjacent room (air temperature 18–20°C). Participants reported for study measures at 0800, following a breakfast of their choice, having received written information in advance to promote adequate hydration. All participant measures were completed by 1300 the same day. Alcohol and caffeinated beverages were not permitted to be consumed in the 24 h beforehand.

Following arrival and first void, urine specific gravity was determined from Multistix (10SG, Siemens, Munich, Germany). Individuals with a specific gravity ≥1.020 were required to drink water sufficient to ensure adequate hydration. Once specific gravity <1.020 was achieved, blood was sampled in the resting recumbent position from an indwelling venous catheter established in the antecubital fossa before and immediately after each of the VO_2_peak assessments and HTA. Centrifugation was performed immediately (plasma) or after separation by clotting (serum), after which serum and plasma were promptly frozen to minus 80°C prior to transfer for off‐site analysis.

Once participants were shown to be hydrated, body composition was estimated by bioelectrical impedance (Body Stat Ltd., Isle of Man, UK), height was measured by stadiometer (Leicester Height Measure, Seca Ltd., Birmingham, UK), and body surface area was calculated according to Du Bois and Du Bois (Bois & Du Bois, 1916), using nude body mass collected immediately prior to HTA (Sartorius, Epsom, UK) and after toweling upon chamber exit at the conclusion of HTA.

At least 60 min after exercise testing, synACTHen (Mallinkrodt Pharmaceuticals plc, Dublin, Ireland) was administered intravenously in a dose of 250 mcg (heat illness cases only). Blood was further sampled from the same indwelling catheter in the seated position immediately prior to injection and at 30 and/or 60 min afterwards.

### 2.3 Heat Tolerance Assessment

Dry and wet bulb temperatures in the chamber were set to 34°C and 24°C (relative humidity 44%).

Participants wore shorts, T‐shirt, and trainers for the determination of VO_2_peak, which was conducted as an incremental running test to volitional exhaustion. Fans were placed in front of the treadmill in order that a wind speed of 1.9 m.s^−1^ was generated. Expired gas was collected, continuously analyzed, and recorded using an on‐line system (CPET, Cosmed, Rome, Italy). Heart rate and electrocardiogram were monitored continuously throughout (Polar Electro, Finland and Welch Allyn, Oregon, USA).

After resting outside the environmental chamber for at least 45 min, the volunteers were then instrumented for skin temperature (not reported) and self‐inserted a rectal probe (Variohm‐Eurosensor Ltd., Towcester, UK) Shorts were removed, and trousers and a jacket, both standard military issue, were donned. Participants then re‐entered and undertook HTA, consisting of three continuous phases (Box 1). During treadmill walking, further measurements were made of expired air. Rectal temperature was monitored (Measurement Specialities Inc., Ireland) throughout. Individuals were considered to demonstrate normal thermoregulation and to be heat tolerant if a plateau occurred in rectal temperature within 90 min. The rationale for this approach has been explained previously (House et al., 2023).

BOX 1 **Heat Tolerance Assessment Protocol**

Phase 1 (0–30 min): Volunteers carried a 14–16 kg rucksack and walked on the treadmill with the carried load, speed, and gradient set to elicit a work intensity equivalent to 60% V̇O_2_peak. After 5 min, expired gas was analyzed to ensure the correct work intensity was achieved, and the speed and gradient were adjusted if necessary. The speed and gradient then remained the same for the rest of the test.

Phase 2 (30–45 min): At 30 min, the rucksack and jacket were removed.

Phase 3 (45–90 min): At 45 min the T‐shirt was removed. Volunteers continued to walk until 60 min and were then stopped if a plateau (i.e., two consecutive readings the same) or fall in rectal temperature occurred; if rectal temperature was still rising, the volunteer continued until a plateau occurred or 90 min had elapsed.

Both VO_2_peak testing and HTA were performed without drinking, with participants encouraged to consume water between assessments.

### 2.4 Biochemical measures

Serum and plasma were analyzed at a supra‐regional endocrine biochemistry service. Plasma was assayed for copeptin (Brahms Kryptor, ThermoFisher) and in‐house liquid chromatography/tandem mass spectrometry quantified normetanephrine and metanephrine levels for cases. Serum was assayed by ion selective electrode for electrolytes (Cobas ISE, Roche, Basel, Switzerland), by enzymatic assay for creatinine (Cobas c702, Roche, Basel, Switzerland), by freezing‐point depression for osmolality (3300 micro‐osmometer, Advanced Instruments Inc., Norwood MA, USA) and by chemiluminescent assay for cortisol (ADVIA Centaur, Siemens, Munich, Germany; detection limits 13.8 to 2069 nmol.L^−1^); and sweat was assayed for the same analytes, except cortisol, using the same methods.

For the serum of heat illness patients only, serum aldosterone was measured by chemiluminescent assay (Immunodiagnostic Systems Ltd., Tyne & Wear, UK; detection limits 103–3300 pmol.L^−1^) and in‐house liquid chromatography/tandem mass spectrometry quantified androstenedione, dehydroepiandrostenedione‐sulphate (DHEAS), testosterone, 17‐hydroxyprogesterone, 11‐deoxycortisol, 21‐deoxycortisol, and 11‐deoxycorticosterone (respective upper and lower reporting limits and inter‐assay coefficients of variation: 0.7–100 nmol.L^−1^ and 7.2%; 0.1–40 μmol.L^−1^ and 4.7%; 0.4–100 nmol.L^−1^ and 6.5%; 1–500 nmol.L^−1^ and 6.6%; 3–100 nmol.L^−1^ and 5.1%; 3–100 nmol.L^−1^ and 5.4%; 1–100 nmol.L^−1^ and 5.2%;). Due to challenges with phlebotomy and sample processing, only 11, 12, and 13 sets of complete results from synACTHen testing (0, 30, and 60 min) were available in the final analysis of 16 nHI participants.

Genetic testing, performed at a regional specialist clinical centre, was by the “CYP21A2 Common 8 Mutation Panel”, plus multiplex ligation‐dependent probe amplification (MLPA) analysis for CYP21A2 dosage. Pyrosequencing was employed to test for point mutations of c.89C > T p.(Pro30Leu), c.290‐13C > G; c.515 T > A p.(IIe172Asn); c.841G > T p.(Val281Leu), c. 952C > T p.(Gin318Ter); c.1066C > T p.(Arg 356Trp), and a CYP21A2 chimeric deletion/conversion encompassing exons 1–3. The c.329_336delGAGACTAC mutation was tested for by fluorescent PCR. Using the P050 CAH MLPA kit (MRC, Holland), the copy number of exons 1,3,4, 6, and 7 of the 21OHD gene was quantified. Bidirectional Sanger sequencing was performed on the promoter region and exons 5,6 and 7 of CYP21A2. Genetic testing of first degree relatives was out with the approvals and scope of the study, such that quantification was restricted to the number of exons and relevant mutations present, rather than number of affected chromosomes.

### 2.5 Statistics

The sum of 17‐hydroxyprogesterone and 21‐deoxycortisol was expressed relative to cortisol as (17OHP + 21DF)/F. For statistical purposes, all steroid values below the sensitivity limit for the particular assay were arbitrarily considered equal to the detection level divided by the square root of two (thereby defining an average number for all undetectable values). Normality of results (parametric versus nonparametric distribution) was assessed by D'Agostino and Pearson test. For statistical comparisons of nHI versus Controls, experimental groups were treated as independent variables. *T*‐tests were applied to compare parametric data and the Mann Whitney *U* test for nonparametric test. Data with repeated measures were analyzed using a general linear mixed model analysis of variance (ANOVA) for repeated measures, with group as the between‐subject factor and time as the within subject measurement. Mauchly's test was undertaken to determine if the assumptions of sphericity were met; if the assumptions were violated, the Greenhouse Geisser correction was used, which amends the degrees of freedom. Levene's test was used to check the homogeneity of variances. Post hoc comparisons were made by *t*‐tests with Bonferroni correction. Data that were nonparametrically distributed were analyzed using the Kruskal‐Wallis test, and post hoc comparisons were made using the Mann–Whitney *U* test. Linear relationships were explored using Pearson (parametric data) or Spearman rho (nonparametric). A value of *p* < 0.05 was considered statistically significant.

## 3 RESULTS

Dry, wet bulb, and globe temperatures in the climatic chamber were 35.0 ± 0.6, 24.2 ± 0.4, and 34.9 ± 0.5°C, producing a WBGT index of 27.4 ± 0.4°C, with relative humidity of 41 ± 2%. Table 1 displays anthropometric and performance data for heat illness cases (nHI and hHI) and controls, all of whom were male and enrolled in either the British Army (both hHI, 13 nHI cases, all controls) or Royal Marines (three nHI Cases). The proportion of these groupings comprising non‐White personnel was <10%, in aggregate across the United Kingdom military during the years studied. This was reflected in the vast majority of study participants (>90%) being of White, Northwest European descent, with no obvious representation of groups at high risk of NCCAH (e.g., Eastern European Jewish, Mediterranean, or Yupik heritage).

**TABLE 1:** Mean (SD) characteristics and VȮ2 peak data for the Patient and Control Groups at the point of sampling for study measures.

	hHI1	nHI (*n* = 16)	Control group (*n* = 16)	*p*‐Value
Age (years)	40	27 (6)	27 (6)	0.990
Height (m)	1.77	1.79 (0.06)	1.80 (0.08)	0.704
Body mass (kg)	91.2	81.2 (6.8)	81.8 (12.0)	0.854
Body surface area (m^2^)	3.63	2.00 (0.11)	2.01 (0.17)	0.814
Body fat (%)	24.3	13.3 (2.6)	17.6 (5.5)^a^	0.009
Lean body mass (kg)	69.0	69.1 (5.6)	67.2 (9.3)	0.814
Absolute VȮ_2_max (L.min^−1^)	3.63	4.50 (0.71)	4.39 (0.63)	0.628
Relative VȮ_2_max (mL.kg.min^−1^)	39.8	56.2 (9.8)	53.9 (6.1)	0.436

^a^ Significant difference, nHI versus Controls.

### 3.1 nHI participants and controls

Of 16 nHI cases, one had been assessed 4 months previously in the service and was thermally intolerant; thus, he was enrolled in the study as a participant reattending for his second HTA, which he passed. For the other 15 volunteers, this was their first assessment; of these, four failed HTA and were required to return for subsequent assessment (conducted outside the study protocol). Incapacity under thermal stress had arisen from a mixture of exertional, non‐exertional, and intermediate mechanisms.

**FIGURE 1:**
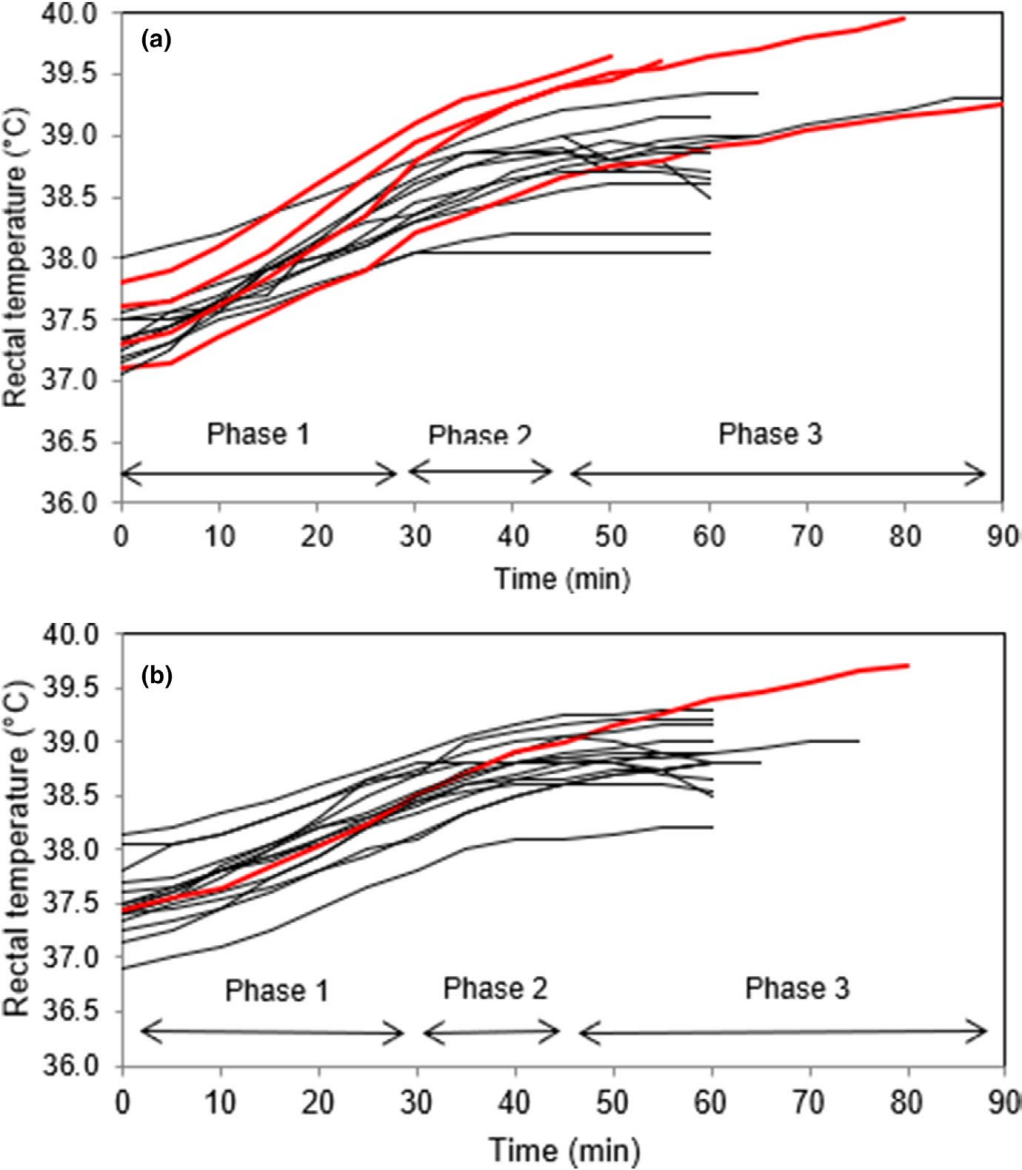
(a) Individual rectal temperature for the normonatremic heat illness cases in response to the Heat Tolerance Assessment. Lines in red are the volunteers in which the anticipated physiological plateau was not achieved. (b) Individual rectal temperatures Controls in response to Heat Tolerance Assessment. Lines in red are the volunteers in which the anticipated physiological plateau was not achieved.

**FIGURE 2:**
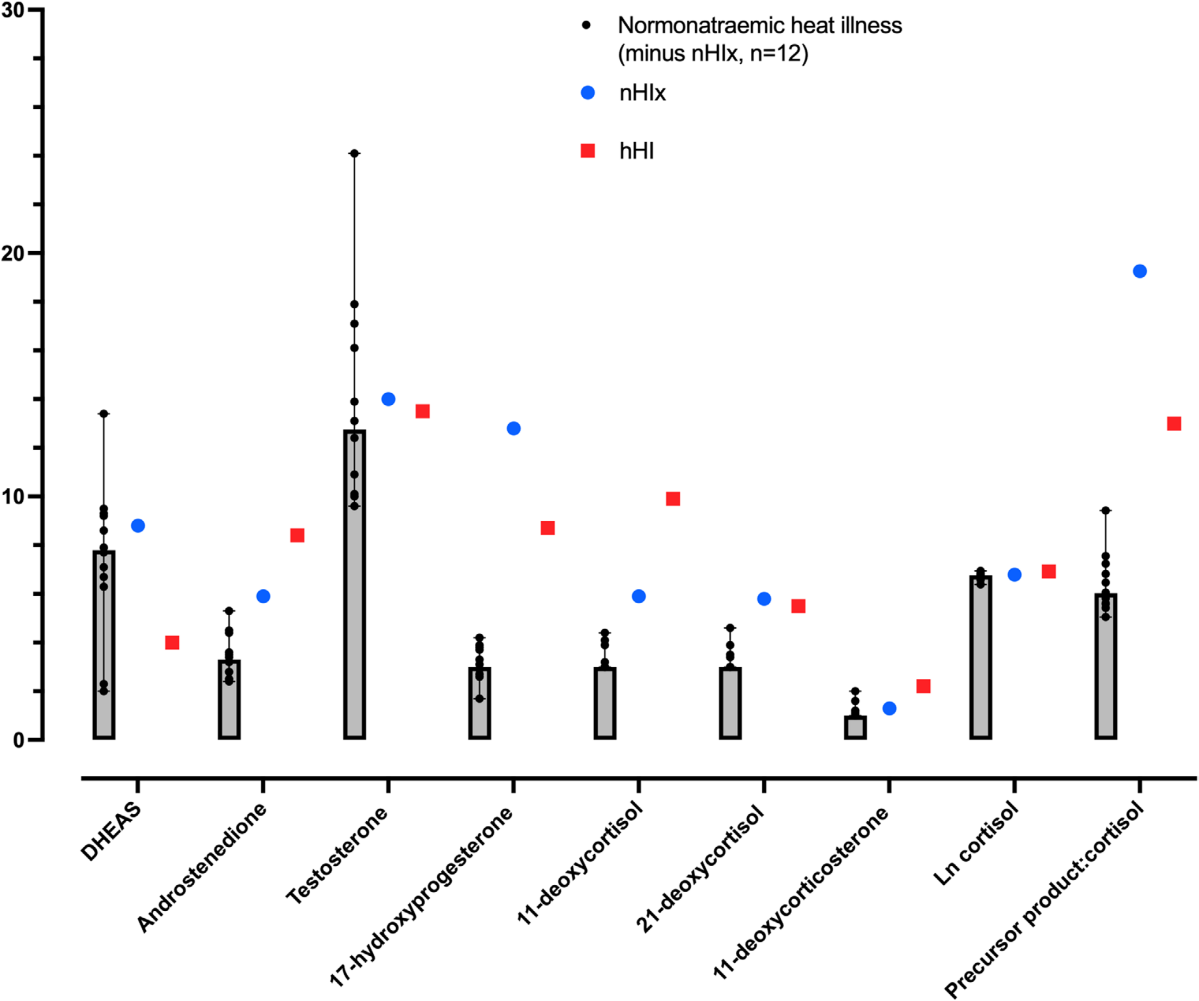
Serum values (median, range) of steroidogenesis products in heat illness cases, assayed from the 60 min point in the short synACTHen test. Complete data are available for 13 of 16 normonatremic cases with 60 min values successfully resulted, of whom nHIx was found to show a biochemical phenotype of 21‐hydroxylase deficiency; and hHI1, one of two hyponatremic cases genetically proven to be affected by 21OHD. DHEAS, dehydroepiandrosterone sulphate.

Data showing HTA performance for nHI and controls are displayed in Figure 1. Sweat rate did not differ between nHI and controls. There was an effect of time, but no main effects of group (nHI vs. cases) or interaction for rectal temperature and heart rate. Table 2 shows the results of blood sampling in relation to HTA. Available steroid hormone responses 60 min following the administration of short synACTHen are displayed in Figure 2; these are also provided alongside baseline and 30 min values, plus unstimulated laboratory reference values, in Table S2.

**TABLE 2:** Biochemical results for hHI1 and nHI patients, and Controls. Data are shown as mean (SD) or median (min to max). Significant differences (*p* < 0.05) in comparison of nHI and Controls.

	hHI1			nHI			Controls		
Analyte (reference range)	Pre	Post V̇O_2_peak	Post HTA	Pre	Post V̇O_2_peak	Post HTT	Pre	Post V̇O_2_peak	Post HTA
Plasma copeptin^a^ (1–12) pmol.L^−1^	4.0	5.9	14.1	4.1 (1.8, 29.1)	11.4 (3.2, 57.1)	18.0 (1.2, 31.9)	4.3 (1.7, 11.0)	7.8 (3.5, 37.9)	14.2 (2.9, 31.2)
Serum sodium (135–145) mmol.L^−1^	139	140	139	141 (2)	140 (2)	141 (2)	141 (1)	140 (1)	141 (1)
Serum potassium^a^ (3.6–5.2) mmol.L^−1^	4.7	4.5	4.4	4.3 (0.5)	4.2 (0.3)	4.7 (0.5)	4.4 (0.4)	4.1 (0.2)	4.5 (0.3)
Serum creatinine^a^, ^b^, ^c^ (60–100) umol.L^−1^	95	100	105	94 (11)	100 (15)	116 (20)	77 (10)	93 (9)	93 (12)
Osmolality^a^, ^c^(275–95) mOsmo.kg‐^1^	287	304	293	293 (5)	296 (5)	293 (5)	297 (4)	305 (20)	297 (6)
Cortisol^a^, ^c^ (140–690) nmol.L^−1^	247	200	428	432 (148)	412 (120)	600 (242)	291 (94)	345 (163)	408 (258)
Aldosterone^a^ (55–250) pmol.L^−1^	169	334	907	134 (59)	548 (252)	1368 (710)	–	–	–
Plasma metanephrines (<510) pmol.L^−1^	317	441	560	291 (85)	496 (191)	472 (188)	–	–	–
Plasma normetanephrines (<1180) pmol.L^−1^	672	1470	2190	468 (135)	1057 (425)	1610 (574)	–	–	–
Salivary cortisol (5–46) nmol.L^−1^ (9 am)	1.2	–	1.6				6.9 [4.2, 19.4]	6.7 [4.4, 10.0]	4.2 [3.8, 19.2]
Salivary cortisone (18–47) nmol.L^−1^ (9 am)	10.8	–	23.6				18.8 [14.3, 25.8]	26.8 [18.4, 32.4]	31.2 [27.0, 47.2]
Urine osmolality mosm.kg^−1^	750	–	–	570 (308)			587 (298)		
Urine sodium mmol.L^−1^	102			84 (62)			80 (53)		
Urine K mmol.L^−1^	69			38 (22)			47 (27)		
Urine Cr umol.L^−1^	23.7			12.8 (9.6)			10.1 (6.4)		
Urine Cortisol nmol.L^−1^ (collected from Pre to Post HTA)	19						117 (71)		

Abbreviations: hHI1, hyponatremic heat illness case 1; HTA, heat tolerance assessment; nHI, normonatremic heat illness cases.

^a^ Main effect of time.

^b^ Interaction between time and Group.

^c^ Main effect of Group.

In one nHI case (nHIx) who had passed HTA, stimulated (17OHP + 21DF)/F of 20.5 exceeded that observed in hHI1 and was above the threshold reported for CYP212A heterozygote status (>13.31^1^) (Ng et al., 2023), though hyponatremia had not been documented in this patient's medical records. Further chart review revealed a medical history positive for recurrent episodes of transient loss of consciousness during military training, predating his episode of heat illness, which occurred on the final exercise of the 32‐week Commando selection course. He had been investigated through a cardiology service, with no abnormalities preventing return to duty found. Serum potassium was 5.7 mmol/L (normal range <5.4 mmol.L^−1^) when sampled shortly after one collapse, without evidence for hyponatremia, hypoglycemia, renal embarrassment, or artifact from hemolysis. Mild eosinophilia was present in all collapse‐associated blood sampling. Inclusion of individual performance and biochemical data relating to this individual did not significantly impact any of the reported relationships described for nHI summary data.

### 3.2 hHI participants

Of the two patients with hHI who were recalled and recruited for the study, both gave consent to the use of their data from during their military Service—including prior HTA results—and to contemporary participation, having each left the military. However, despite material support to attend, only one individual (hHI1) was able to complete contemporary HTA for dynamic biochemical profiling, with hHI2 unable to overcome the psychological stress associated with further exposure to the climatic chamber and assessment process. An updated account of the personal and professional experiences of hHI1, from the interval since the presenting episodes, is provided at Box 2, demonstrating a significant impact of the episodes on his well‐being also.

BOX 2 **Testimony of Hyponatremic Heat Illness Case hHI1**

I deployed to Iraq to be part of the Quick Reaction Force, taking part in vehicles and (dismounted) patrols. I was carrying an ankle injury going into the tour and had lost a bit of fitness, so I was (medically) downgraded, but still deployed. About three and a half months in, I was out on patrol and became confused… the blokes said I just wandered off… I just remember doing my own thing; I even left my weapon in camp apparently.

I was admitted to the field hospital for about 2 weeks until my salt levels had got back to normal and made a good recovery. When I redeployed out on the ground, I had a medic to keep an eye on me, make sure I was taking enough salt, and give me Dioralyte…. But I had another similar episode. This time, because I had been encouraged to wear a T‐shirt rather than a U‐back (which had previously been covering my arms), I noticed that my arms had gone all white, all crystallized.

I still completed my operational tour, doing night sentry for the rest of it rather than going out and about in the day. I felt fine doing my assessment in the heat chamber, though it took nearly 2 years to get there.

I went to Canada for an exercise on the Prairie and had a similar episode—again, a medic was assigned to watch me. I was in an armored vehicle and had been told to stay inside it the whole time, including sleeping, with the air conditioning on. The other guy with me was out and about round the vehicle in the heat while I stayed inside. A medic had been assigned to watch me again. Three weeks in, the hatch had to be opened (on orders) and the air conditioning switched off to stop the dust from being sucked in and wrecking things. We spent half an hour like that, with the temperature rising. Then I got sick and started vomiting. My colleague was fine. I went off to get medically assessed and they thought I was good to carry on, though I kept the hatch closed like I had been told to after that.

After getting heat illness, they tried me on runs and marches but had to assign two PTIs to check my temperature and heart rate weren't getting too high. I get called “Pink and Fluffy”, no one will touch me because of the risk of heat illness… My career has definitely suffered as I can't do the marches on promotional courses or deploy to a hot exercise or operational tour.

Since then, I have been advised not to attempt military fitness tests and told to observe my own program. I am back on re‐conditioning now (after 10 years). The funny thing is, before I joined the Army (aged 26), I was a holiday rep I Turkey for a year and coped fine—I was spending the days by the pool and perhaps rehydrated better.

**FIGURE 3:**
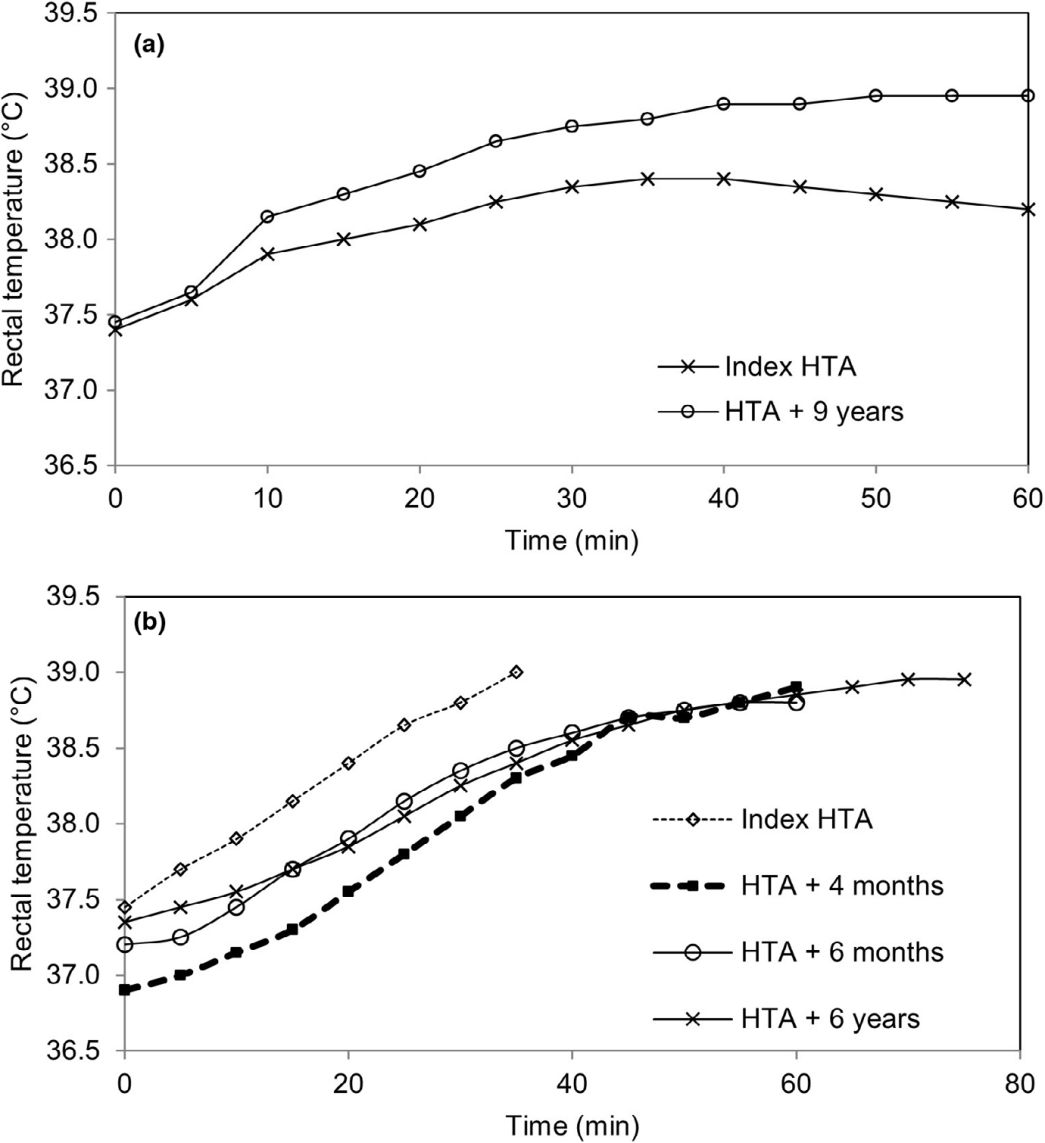
Rectal temperature with HTA in (a) hHI1 (plateau in both assessments indicative of Pass) and (b) hHI2 (plateau in latter assessments indicative of Pass).

Results of serial HTA for hHI1 and hHI2 are shown in Figure 3. Pooled (*n* = 32, nHI plus controls) results for sweat rate (1.26 [0.83, 2.25] L.h^−1^) and sweat rate adjusted for body surface area (610 [442, 1130] mL.m^2^.h^−1^) compared with contemporary study results in hHI1 of 0.62 L.h^−1^ and 295 mL.m^2^.h^−1^ and historic values from serial clinic assessments of 1.19 L.h^−1^ and 608 mL.m^2^.h^−1^; and with corresponding historic values of 1.32, 1.01, 1.00, 0.85 L.h^−1^ and 697, 543, 537, 440 mL.m^2^.h^−1^ in the four HTAs performed by hHI2. Historic anthropometric and V̇O_2_max data, and HTA details for the two case studies are given in Table S1.

Completing HTA as a contemporary study participant, hHI1 showed thermoregulatory responses consistent with a pass. Aldosterone was assayed in relation to HTA only for nHI; however, in hHI1, additional measures were made for short synACTHen test timepoints 0, 30, and 60 min. These results were 533, 807, and 689 pmol.L^−1^, respectively, which compared with 169, 334, and 907 pmol.L^−1^ (rested baseline, post‐VO_2_peak and post‐HTA) from the HTA performed beforehand.

Genotyping of hHI1 revealed heterozygosity for both the pathogenic CYP21A2 mutations c.515 T > A p.(IIe172Asn) and c.841G > T p.(Val281Leu). In hHI2, multiple heterozygous mutations of known pathogenic nature were evident upon analysis of the gene, its promoter region, and exons 5 to 7. Both individuals were referred for genetic counseling in the UK civilian healthcare system.

Full histories of the clinical, occupational, and assessment circumstances relating to their episodes of incapacitation are provided in the Appendix S1 online.

## 4 DISCUSSION

In our study, 21OHD was implicated in three of 18 military heat illness cases recruited from a specialist occupational assessment service. None of these cases had previously been diagnosed with an adrenal disorder, and recognition that steroidogenesis may be suboptimal required specialist biochemical and genetic investigations, beyond the orthodox determination of adrenal reserve usually achieved with synACThen testing. Despite including one of these individuals—who was not initially suspected of 21OHD—in the analysis of our normonatremic patient group (nHI), these other heat illness cases did not obviously manifest inadequate adrenocortical responses to standardized exercise in the heat, either in serum aldosterone or total cortisol; indeed the latter was actually higher than in the control group of healthy participants lacking a history of heat illness.

Taken together, these findings implicate a relative deficiency of adrenal reserve in only a minority of heat illness cases, and perhaps only under conditions of longer length and greater cumulative stress than they encountered in the HTA. The finding that total cortisol responses were not lower versus controls in the nHI patient group, but rather higher, does not appear to relate to any relative increase in physiological strain with the HTA, as neither rectal temperature nor heart rate showed an interaction (Time × Group) across the assessment. However, with the hHI cases all undergoing the HTA as part of a clinical assessment used to inform an occupational decision on return‐to‐duties or fitness for continued military service, this difference may partly reflect increased psychological stress associated with the exposure, which was not itself measured in our study. This finding does not, in any case, indicate a need for more global concern for adrenal reserve in heat illness cases per se.

The suggestion of relative adrenal insufficiency was more immediately obvious in the two cases presenting with low serum sodium levels at the time of incapacity (hH1 and hH2). Both of these individuals were deemed likely to be compound heterozygotes for CYP212A in cause of nonclassic CAH, with alternate mutations donated from their respective mothers and fathers. In the solitary member of the pair able to undergo full biochemical categorization (hHI1), HTA was associated with relatively low free cortisol responses. Additionally, a third case—for whom hyponatremia had not been documented, but where features of adrenal insufficiency (hyperkalemia and persistent eosinophilia) preceded and coincided with heat illness—evidenced a biochemical phenotype convincing for 21OHD. Specifically, 30 min after the administration of synACTHen, he showed 21‐deoxycortisol of 4.8 nmol.L^−1^ (compared with 2.2 nmol.L^−1^ in hHI1, and values >1.0 nmol.L^−1^ reported to discriminate genetically‐proven 21OHD vs. controls with 100% sensitivity and 90.5% specificity (Ng et al., 2023)) and, at 60 min post‐stimulation, 17OHP of 12.8 nmol.L^−1^ (8.7 nmol/L in hHI1; respective sensitivity and specificity 100% and 80.4% for levels >8 nmol.L^−1^).

Nevertheless, given time to recover from their acute clinical episodes, these three patients did not show consistent impairment of cortisol responses to dynamic provocation, and total serum cortisol levels also appeared robust in the two for whom aldosterone measures were available. None showed evidence of sweat rates or sodium wasting disproportionate to controls. Furthermore, while shunting into the androgen pathway was evident in elevated precursor‐to‐product ratios, physical strain with HTA was not dissimilar to the other populations studied, and did not associate with potential acute compensatory mechanisms, for example, substantially higher excursions in circulating copeptin or catecholamine metabolites. All three individuals showed the ability to pass procedural screening for ongoing vulnerability to occupational heat stress at some point in their individual clinical trajectories, achieving short‐term thermal equilibrium during tailored HTA, under conditions severe enough to cause test failure in five other heat illness cases without impaired pathways.

It appears, therefore, that acute provocatory testing was inadequate to recapitulate the potential pathology underlying their prior episodes of incapacity. This is congruent with the “compensated partial aldosterone insufficiency” described previously for NCCAH, which was reported to associate with no relative impairment of hemodynamic tolerance acutely, yet caused the authors to doubt “whether these patients could adapt to more severe and prolonged dehydration.” (Kamenický et al., 2019) The two genetically proven cases of NCCAH described in the present work developed heat illness while subject to extreme temperatures in Iraq and Afghanistan, in a period of mass military deployment, during which 18% of 116 referrals to the heat illness service described were complicated by serum sodium levels <130 mmol.L^−1^ (unpublished audit data). Sweat sodium losses failing to keep pace with replenishment across time—gradually but progressively, as might be seen with a mild defect in the mineralocorticoid pathway (Ladell & Dhephard, 1962)—would accord with the longer chronicity of symptoms in military heat illness patients who presented in Iraq with low blood sodium levels, whose overall symptom severity was no worse than in normonatremic cases (Coleman et al., 2022; World & Booth, 2008).

Conversely, nHIx—who manifested, on formal testing, a biochemical phenotype congruent with 21OHD—was incapacitated in the temperate climate of the United Kingdom during the final stage of recruitment to the elite Royal Marine Commando force. This selection course is recognized internationally as one of the longest and most arduous of its kind, and case record review revealed that he was carrying a substantial accrued burden of musculoskeletal injuries and receiving “analgesia.” Under this scenario—and especially should he have been taking agents known to antagonize the renin‐angiotensin‐aldosterone system, such as nonsteroidal anti‐inflammatory agents (Fitzpatrick et al., 2024)—thermally mediated collapse may have been precipitated even in a cooler and less consistently severe climate.

Strengths of our study include the use of HTA conducted at an intensity tailored to the participants' individual aerobic fitness. Gearing the HTA to individual performance should help to mitigate changes in aerobic fitness and factors affecting thermoregulation over serial assessments. It is tempting to infer from the higher body fat in controls a reduced capacity to store body heat and limit core temperature rise. This would help to account for the lack of interaction in observed rectal temperatures between groups with HTA, despite a higher rate of thermal responses indicating a “fail” in the nHI group. However, we acknowledge the limited accuracy of bioimpedance and would not seek to draw further conclusions of this nature to explain any differences between groups based on body composition.

Developments over the extended period across which the study was executed meant that salivary cortisol only became readily accessible for the control cohort and hHI1's eventual study participation, whereas nHI cases enrolled in the HIC beforehand lacked this measure. Similarly, in light of apparent adequacy in normetanephrine and aldosterone responses reported for cases as the study progressed, these results were not prioritized for the control cohort. Limitations in the completeness of biochemical data relating to synACTHen testing in nHI related to difficulties in sampling from the indwelling intravenous catheter, placed before the exercising components of the assessment, which showed reduced patency in the post‐HTA phase, resulting in hemolysis and inadequate sample volumes in some participants. This may have related to an increased propensity for coagulation and clotting tendency, as reported with strenuous exercise (Smith et al., 2004).

Lastly, future research would doubtless benefit from the inclusion of more hyponatremic cases of heat illness, with the present work being limited in its ability to recruit cases by falling troop deployments to the more extreme heat of enduring campaigns in Iraq and Afghanistan, which saw fewer such referrals to the heat illness clinic. Achieving adequate statistical power for a more definitive comparison of NCCAH rates between hyponatremic and normonatremic heat illness cases would require many more participants (perhaps 32 in each group, for 80% power based on anticipated prevalence). Hence, this line of enquiry may remain hypothesis‐generating until such a time as vulnerable populations travel into extreme heat in greater numbers, or a more networked, multicentre approach is established.

## 5 CONCLUSIONS

Among patients presenting with features of relative hypoadrenalism to a specialist heat illness clinic, we have demonstrated the presence of characteristic defects in steroidogenesis arising from genetic mutation in CYP21A2. Alongside excessive water intake, unreplaced sodium losses in sweat, and nonsteroidal anti‐inflammatory use, the potential for 21OHD from NCCAH to contribute to exertional hyponatremia and heat illness should be considered, especially when thermal stress is imposed for prolonged periods.

In 21OHD, short format HTA may not accurately reflect future risk from sustained heat stress, and tools to better define and quantify this risk should be sought. In addition to considering genetic testing as part of the diagnostic work‐up post‐incapacity, screening in advance of sustained exposure to extreme environmental heat stress should be considered for at‐risk populations.

## FUNDING INFORMATION

Funding was provided by the Drummond Committee of the Royal Army Medical Corps charity and through a Society for Endocrinology Early Career grant, both awarded to MJS.

## CONFLICT OF INTEREST STATEMENT

There are no conflicts of interest to declare.

## ETHICS STATEMENT

Ethical approval was obtained from the UK Ministry of Defence Research Ethics Committee (856/MODREC/2018), and the study was conducted in accordance with the Declaration of Helsinki, with written informed consent provided by all participants.

## Supporting information


Appendix S1.

## Data Availability

Data available on request.
