# Supplementary material for: Adrenal steroid hormone responses to exercise under thermal stress: Potential role for nonclassic congenital adrenal hyperplasia in heat illness susceptibility
Source: Physiol Rep. 2025 Mar 20;13(6):e70272. doi: 10.14814/phy2.70272 (PMC11923862; doi:10.14814/phy2.70272)
Supplement: Supplementary file 1 — Appendix S1. [file PHY2-13-e70272-s001.docx]

**Supplementary materials**

Hyponatraemic heat illness case details – hHI1

*History of heat illness incidents.* hHI1 suffered two episodes of heat illness when working as a tank driver in Iraq and living in a desert Forward Operating Base. He reported drinking large volumes of water and consuming issued rations. He was admitted to hospital with a 3-day history of fever, lethargy, weakness, muscle fatigue, dizziness and a dry cough. Bloods revealed hyponatraemia, hypokalaemia with a raised bicarbonate; he was treated with a high salt solution and rest. After 24 hours his biochemistry returned to normal, and after two weeks of light duties he was discharged back to his unit. Despite maintaining good fluid and high salt intake he suffered a second episode with similar symptoms approximately 3 weeks later. Following recovering hHI1 remained deployed but was confined to the Central Operating Base.

He was referred to the HIC for assessment two years later, and passed Heat Tolerance Assessment (HTA). He was further assessed by HTA nine years later, for purposes of the present work, and passed HTA again. He had suffered a further episode of incapacity associated with exposure to environmental heat training on the prairie in Canda in the interim (see Box 2 main article), which was not investigated formally through the HIC.

Hyponatraemic heat illness case details – hH2

5.2.1 hHI2 had been in Iraq for approximately five days and presented to the field hospital with a 3 day history of nausea, vomiting and abdominal cramps which developed whilst doing two hour shifts guarding oil installations. He reported that he was dehydrated and on admission that he had been anuric for 72 hours, his bloods on admission showed acute renal failure, urea was 31.6 mmol.L^-1^, creatinine was 472 umol.L^-1^ and sodium was 114 mol.L^-1^. He was admitted to HDU overnight and given vigorous fluid replacement, and became polyuric within 4 hours and his urea and electrolytes returned to normal within 24 hours; an ultrasound of his kidneys was unremarkable.

Three years later, during summer months, he reported to the local medical facility as he felt unwell during a driving course (ambient temperature ~ 30 °C) but did not collapse. The following month, he collapsed at home (UK) after guard duty, lost consciousness for ~ 30 s with possible convulsions. He was admitted to hospital and investigated for a possible pulmonary embolism, the diagnosis was of chest pain of unknown cause.

Seven years later, while deployed in southern Afghanistan, hHI2 experienced a gradual then rapid deterioration over a few days. He reported feeling weak, lethargic, and ill and vomiting, he also had muscle cramps and was unable to continue working. He again required hospitalisation for acute kidney injury.

His outcomes at the HIC included failed HTA within eight months of his initial presentation in Iraq (failed again four months later; passed after a further two months), and a pass following his deployment to Afghanistan.

Summary data for hyponatraemic heat illness cases (hHI1 and hH2)

Supplementary Table 1. Anthropometric and V̇O_2_max data, speed and gradient adopted for Heat Tolerance Assessment (HTA) in hHI1 and hHI2; percentage of V̇O_2_max and metabolic heat production (MHP) for each HTA Phase; Maximum rectal temperature, rates of rise of rectal temperature and sweat rates.

|  | | hHI1 | | hHI2 | | | | | |
| --- | --- | --- | --- | --- | --- | --- | --- | --- | --- |
| Date | | Index HTA  **Pass** | HTA + 9 y  **Pass** | Index HTA  **Fail** | HTA + 4 months  **Fail** | | HTA + 6 months  Pass | HTA + 6 y  **Pass** | |
| Age (years) | | 31 | 40 | 25 | 26 | | 27 | 32 | |
| Height (m) | | 1.77 |  | 1.77 |  | |  |  | |
| Body mass (kg) | | 78.5 | 91.2 | 73.2 | 70.1 | | 71.1 | 77.6 | |
| Absolute V̇O_2_max (L.min^-1^) | | 2.91 | 3.63 | 3.69 | 3.78 | | 3.57 | 3.60 | |
| Relative V̇O_2_max (mL.kg.min^-1^) | | 37.0 | 39.8 | 50.5 | 53.9 | | 50.2 | 46.4 | |
| Speed (kph) & gradient (%) | | 5.0 & 5.0 | 4.8 & 4.8 | 6.0 & 6.5 | 6.0 & 5.7 | | 6.0 & 6.0 | 5.3 & 5.0 | |
| % V̇O_2_max Phase 1 | | 60 | 60 | 61 | 59 | | 59 | 60 | |
| % V̇O_2_max Phase 2 | | 50 | 55 |  | 52 | | 54 | 44 | |
| % V̇O_2_max Phase 3 | | 46 | 56 |  | 49 | | 55 | 53 | |
| MHP (W.kg^-1^) Phase 1 | | 6.8 | 7.5 | 9.0 | 9.7 | | 9.0 | 8.6 | |
| MHP (W.kg^-1^) Phase 2 | | 5.5 | 6.7 |  | 8.4 | | 8.2 | 6.1 | |
| MHP (W.kg^-1^) Phase 3 | | 5.1 | 6.9 |  | 7.9 | | 8.3 | 7.6 | |
| Maximum rectal temperature (°C) | 38.4 | | 38.9 | 39.0 | 38.9 | 38.8 | | | 38.9 |
| Rate of rise of rectal temperature 10 to 30 min (°C.hr^-1^) | 1.4 | | 1.8 | 2.7 | 2.7 | 2.7 | | | 2.1 |
| Rate of rise of rectal temperature 30 to 45 min (°C.hr^-1^) | 0.0 | | 0.6 |  | 2.6 | 1.4 | | | 1.6 |
| Rate of rise of rectal temperature 45 to 60 min (°C.hr^-1^) | -0.6 | | 0.2 |  | 0.8 | 0.4 | | | 0.8 |
| Sweat rate (L.hr^-1^) | 1.19 | | 0.62 | 1.32 | 1.01 | 1.00 | | | 0.85 |
| Sweat rate relative to BSA (mL.m^2^.hr^-1^) | 608 | | 295 | 697 | 543 | 537 | | | 440 |

*Fail, but allowable time to demonstrate plateau in physiological response extended by HIC to 90 min, prior to final assessment at + 6 years

Supplementary Table 2. Serum biochemistry from short synACTHen test (0, 30 min and/or 60 min) for hHI1 and nHI. Results are shown as mean (SD). Abbreviations: heat tolerance assessment; hHI1, hyponatraemic heat illness case 1; nHI, normonatraemic heat illness cases.

|  | hHI1 | | | nHI | | |
| --- | --- | --- | --- | --- | --- | --- |
| Analyte  (reference range) | T0 | T30 | T60 | T0 (n=11) | T30 (n=12) | T60 (n=13) |
| Cortisol | 533 | 920 | 1017 | 544 [356, 645] | 805 [749, 872] | 878 [793, 915] |
| Androstenedione  (2.1-10.8) nmol.L^-1^ | 4.9 | 8.2 | 8.4 | 2.9 [2.3, 4.3] | 3.5 [2.8, 5.0] | 3.4 [2.7, 4.5] |
| DHEAS  (2.68-11.0) umol.L^-1^ | 4.7 | 3.9 | 4 | 8.8 [7.0, 9.5] | 8.7 [7.2, 10.0] | 7.9 [6.5, 9.3] |
| Testosterone  (7.9-31.3) nmol^-1^ | 15.6 | 14.7 | 13.5 | 18.3 [12.2, 25.7] | 16.3 [12.0, 19.4] | 13.1 [10.1, 16.6] |
| 17-hydroxyprogesterone (<9.6) nmol.L^-1^ | 2.9 | 8.6 | 8.7 | 1.9 [1.4, 3.0] | 3.2 [2.6, 4.3] | 3.5 [2.8, 3.9] |
| 11-deoxycortisol  (0.7-2.7) nmol.L^-1^ | <3 | 9.1 | 9.9 | 3.0 [3.0, 3.0] | 3.0 [3.0, 3.6] | 3.0 [3.0, 4.0] |
| 21-deoxycortisol  (<0.17) nmol.L^-1^ | <3 | 4.8 | 5.5 | 3.0 [3.0, 3.1] | 3.0 [3.0, 3.9] | 3.0 [3.0, 3.75] |
| 11-deoxycorticosterone (0.7-2.7) nmol.L^-1^ | <1 | 2.1 | 2.2 | 1.0 [1.0, 1.0] | 1.0 [1.0, 1.28] | 1.0 [1.0, 1.25] |
| (17OHP + 21DF)/F  (molar weight) | 8.8 | 13.5 | 13.0 | 7.7 [6.2, 13.3] | 6.1 [5.8, 8.5] | 6.1 [5.6, 7.4] |
